# Propofol-Related Infusion Syndrome: A Bibliometric Analysis of the 100 Most-Cited Articles

**DOI:** 10.7759/cureus.46497

**Published:** 2023-10-04

**Authors:** Sophie Van, Vicky Lam, Kisan Patel, Andrew Humphries, Javed Siddiqi

**Affiliations:** 1 Anesthesiology, California University of Science and Medicine, Colton, USA; 2 Physical Medicine and Rehabilitation, California University of Science and Medicine, Colton, USA; 3 Neurological Surgery, Riverside University Health System Medical Center, Moreno Valley, USA

**Keywords:** systematic review and meta analysis, propofol syndrome, propofol infusion syndrome, medical publications, bibliometric analysis

## Abstract

Propofol-related infusion syndrome (PRIS) is a rare, yet life-threatening sequelae to prolonged administration of the anesthetic propofol in mechanically intubated patients. The condition is characterized by progressive multi-system organ failure and eventual mortality; of note, the predominant characteristics of PRIS involve but are not limited to cardiovascular impairment and collapse, metabolic and lactic acidosis, rhabdomyolysis, hyperkalemia, and acute renal failure. While potent or extended doses of propofol have been found to be the primary precipitating factor of this condition, others such as age, critical illness, steroid therapy, and hyperlipidemia have been discovered to play a role as well. This bibliometric analysis was done to reflect the current relevance and understanding of PRIS in recent literature. The SCOPUS database was utilized to conduct a search for articles with keywords “propofol infusion syndrome” and “propofol syndrome” from February 24, 2001, until April 16, 2023, with parameters for article title, citation number, citation per year, author, institution, publishing journal, and country of origin. PRIS was first defined in 1990, just a year after its approval by the Food and Drug Administration for use as a sedative-hypnotic. Since then, interest in PRIS slowly rose up to 13 publications per year in 2013. Seven papers on the topic were published in *Critical Care Medicine,* six in *Neurocritical Care*, and four in *Anesthesia*. The most common institutions were Mayo Clinic, Northeastern University, and Tufts Medical Center. To our knowledge, this is the first bibliometric analysis to evaluate the most influential publications about PRIS. A majority of the research is case-based, possibly owing to the rarity of the condition. Our research suggests that confounding factors outside the precipitating dosage of propofol may be implicated in the onset and progression of PRIS. This study could therefore bring renewed interest to the topic and lead to additional research focused on fully understanding the pathophysiology of PRIS in order to promote the development of novel diagnostics and treatment.

## Introduction and background

In recent years, bibliometric analysis has emerged as a useful tool for assessing the scientific findings within various fields of research. By quantitatively analyzing publications and their citations, bibliometric studies provide insights into the scholarly impact and knowledge of specific topics [1]. In medical research, bibliometric analyses are valued in examining research trends and highlighting key contributors to the advancement of medical knowledge [2].

An area of interest in anesthesia and critical care medicine is propofol-related infusion syndrome (PRIS). PRIS is a rare but potentially fatal complication associated with the prolonged use of propofol, a widely used intravenous anesthetic agent [3]. First described in the 1990s, PRIS has since gained attention due to its consequences and our limited understanding of the syndrome [4].

Propofol, 2,6-diisopropylphenol, an intravenous anesthetic agent with sedative and hypnotic properties, is a widely used drug in intensive care units and operating rooms. Its rapid onset and short duration of action make it an ideal anesthetic drug [5]. However, despite its widespread use and benefits, concerns regarding the safety of propofol have emerged, particularly regarding the development of PRIS [5-10].

PRIS is characterized by metabolic acidosis, rhabdomyolysis, cardiovascular collapse, and multi-organ failure [3,4,6]. Although the exact pathophysiology of PRIS is poorly understood, it is thought to be influenced by various factors, including cumulative dose, duration of infusion, concomitant medical conditions, and patient-specific factors [6,11]. The identification of these risk factors and the development of effective preventive strategies are essential to mitigate the occurrence and severity of PRIS.

Given the seriousness of PRIS and the need for a comprehensive understanding of this syndrome, a bibliometric analysis can offer valuable insights into the research surrounding PRIS. By systematically analyzing the scientific literature and its associated citations, a bibliometric study can provide a comprehensive overview of the research output, key contributors, influential journals, and emerging trends in PRIS research [12]. Such an analysis can guide future research direction and contribute to enhanced patient care and safety.

In this research paper, we present a bibliometric analysis of the literature on PRIS to explore the evolution of research output and identify influential publications and authors. We aim to provide a comprehensive overview of the scientific landscape surrounding PRIS, which will facilitate a deeper understanding of the syndrome and offer valuable insights for clinicians and researchers.

## Review

Methods

The database SCOPUS was used to search for the top 100 cited articles on PRIS. The keyword-based search focused on “propofol infusion syndrome” and “propofol syndrome” was used. All articles published before August 2023 were included. Document type was limited to articles written in the English language. The results of 165 articles were sorted by the highest number of citations. The top 100 most-cited articles were thoroughly studied. We compiled a list of citation count, year of publication, source journals, affiliated institutions, funding sponsors, authors, and countries contributing significantly to propofol infusion syndrome research. This data sheet was then utilized to generate tables and graphical representations for visualization and comparison. An overview of the articles was also included. Finally, this research paper was written based on the findings of the bibliometric analysis.

Table 1 is an exact replica of our refined SCOPUS database search.

**Table 1:** SCOPUS database search

Propofol Infusion Syndrome Publishing Trends	
Search Phrase	TITLE-ABS-KEY (propofol AND infusion AND syndrome) AND TITLE-ABS-KEY (propofol AND syndrome)
Keyword	Propofol infusion syndrome
Time	All
Inclusion criteria	Document type (Article), Publication stage (Final), Language (English)
Exclusion criteria	None
Results	165
Sort	Cited by (highest)

Results

The list of the top 100 articles on propofol infusion syndrome, organized from most to least cited is shown in Table 2. These articles gained a total of 3856 citations.

**Table 2:** Top 100 most cited articles on PRIS PRIS: propofol-related infusion syndrome

Rank	Title	Citation count
1	Propofol infusion syndrome [13]	364
2	Impaired fatty acid oxidation in propofol infusion syndrome [14]	336
3	The outcome of therapies in refractory and super-refractory convulsive status epilepticus and recommendations for therapy [15]	319
4	A randomized trial for the treatment of refractory status epilepticus [16]	170
5	Incidence of propofol-related infusion syndrome in critically ill adults: A prospective, multicenter study [17]	140
6	Volatile anesthetics versus total intravenous anesthesia for cardiac surgery [18]	133
7	Propofol infusion syndrome: A structured review of experimental studies and 153 published case reports [19]	126
8	Propofol infusion syndrome in patients with refractory status epilepticus: An 11-year clinical experience [20]	122
9	Propofol infusion syndrome in critically ill patients [21]	102
10	Predictors of mortality in patients with suspected propofol infusion syndrome [22]	91
11	Fatal propofol infusion syndrome in association with ketogenic diet [23]	87
12	Propofol infusion syndrome associated with short-term large-dose infusion during surgical anesthesia in an adult [24]	82
13	Adverse drug events associated with the use of analgesics, sedatives, and antipsychotics in the intensive care unit [25]	79
14	Possible pathogenic mechanism of propofol infusion syndrome involves coenzyme Q [26]	56
15	Propofol treatment in adult refractory status epilepticus. Mortality risk and outcome [27]	55
16	Lactic acidosis during propofol-remifentanil anesthesia in an adult [28]	55
17	Toxicity of intravenous anaesthetics [29]	53
18	Refractory and super-refractory status epilepticus - An update [30]	51
19	Long-term outcome of refractory status epilepticus in adults: A retrospective population-based study [31]	50
20	Incidence of propofol infusion syndrome during noninvasive radiofrequency ablation for atrial flutter or fibrillation [32]	45
21	Propofol infusion syndrome: An unusual cause of renal failure [33]	45
22	Death after re-exposure to propofol in a 3-year-old child: A case report [34]	43
23	Propofol and barbiturates for the anesthesia of refractory convulsive status epilepticus: Pros and cons [35]	39
24	Propofol induces a metabolic switch to glycolysis and cell death in a mitochondrial electron transport chain-dependent manner [36]	37
25	Drug-induced acid-base disorders [37]	36
26	Propofol compared with isoflurane inhibits mitochondrial metabolism in immature swine cerebral cortex [38]	34
27	Treatment of pediatric status epilepticus [39]	34
28	Patient-ventilator interaction [40]	34
29	Clinical Practice Guideline for Emergency Department Procedural Sedation With Propofol: 2018 Update [41]	33
30	Systemic complications of status epilepticus - An update [42]	32
31	Survival of propofol infusion syndrome in a head-injured patient [43]	32
32	A case of suspected non-neurosurgical adult fatal propofol infusion syndrome [44]	32
33	Can anesthetic treatment worsen outcome in status epilepticus? [45]	30
34	Refractory status epilepticus: New insights in presentation, treatment, and outcome [46]	30
35	Inborn oxidative phosphorylation defect as risk factor for propofol infusion syndrome [47]	28
36	Effects of anesthetics on mitochondrial signaling and function [48]	28
37	Routine use of recombinant human bone morphogenetic protein-2 in posterior fusions of the pediatric spine and incidence of cancer [49]	27
38	Use of propofol infusion in alcohol withdrawal-induced refractory delirium tremens [50]	27
39	Neuromuscular disease and anesthesia [51]	27
40	Analysis of the delay components in the treatment of status epilepticus [52]	27
41	Intubation and mechanical ventilation of the asthmatic patient in respiratory failure [53]	26
42	Pharmacological management of sedation and delirium in mechanically ventilated ICU patients: Remaining evidence gaps and controversies [54]	25
43	When should sedation or neuromuscular blockade be used during mechanical ventilation? [55]	25
44	Causes of death in status epilepticus [56]	24
45	Use of Propofol-Containing Versus Benzodiazepine Regimens for Alcohol Withdrawal Requiring Mechanical Ventilation [57]	24
46	Is propofol a friend or a foe of the pediatric intensivist? description of propofol use in a PICU [58]	23
47	Effect of a single dose of propofol and lack of dextrose administration in a child with mitochondrial disease: A case report [59]	22
48	Propofol infusion syndrome: A lethal condition in critically injured patients eliminated by a simple screening protocol [60]	22
49	Vasopressors and propofol infusion syndrome in severe head trauma [61]	22
50	Propofol infusion syndrome: Case report and literature review [62]	20
51	Multiple organ failure after an overdose of less than 0.4 mg/kg of colchicine: Role of coingestants and drugs during intensive care management [63]	19
52	The multifaceted care of status epilepticus [64]	18
53	Monitoring biochemical parameters as an early sign of propofol infusion syndrome: False feeling of security [65]	18
54	Propofol Related Infusion Syndrome: Ultrastructural Evidence for a Mitochondrial Disorder [66]	16
55	Is there a relationship between hyperkalemia and propofol? [67]	15
56	Hyperkalemia during surgery: Is it an early warning of propofol infusion syndrome? [68]	14
57	Fatal measles pneumonitis during Hodgkin's lymphoma [69]	13
58	Propofol infusion syndrome heralded by ECG changes [70]	13
59	Fospropofol [71]	13
60	Prevention, treatment, and monitoring of seizures in the intensive care unit [72]	12
61	Experience with the use of propofol for radiologic imaging in infants younger than 6 months of age [73]	12
62	Therapeutic plasma exchange as treatment for propofol infusion syndrome [74]	12
63	Propofol versus thiopental sodium for the treatment of refractory status epilepticus (Review) [75]	12
64	Propofol infusion syndrome during refractory status epilepticus in a young adult: Successful ECMO resuscitation [76]	12
65	Propofol Infusion Syndrome With Arrhythmia, Myocardial Fat Accumulation and Cardiac Failure [77]	12
66	Propofol infusion syndrome in a patient with sepsis [78]	12
67	Pharmacotherapy Pearls for Emergency Neurological Life Support [79]	11
68	Propofol-related infusion syndrome induced by "moderate dosage" in a patient with severe head trauma [80]	11
69	Partial-exchange blood transfusion: An effective method for preventing mortality in a child with propofol infusion syndrome [81]	11
70	Use of pharmaceuticals 'Off-Label' in the neonate [82]	11
71	Behavioral and toxicological effects of propofol [83]	10
72	The Safety of Continuous Infusion Propofol in Mechanically Ventilated Adults With Coronavirus Disease 2019 [84]	9
73	The child with glutaric aciduria type I: Anesthetic and perioperative management [85]	9
74	Comparison of propofol and dexmedetomidine infused overnight to treat hyperactive and mixed ICU delirium: A protocol for the Basel ProDex clinical trial [86]	8
75	Comparison of Rhabdomyolysis Markers in Patients Undergoing Bariatric Surgery with Propofol and Inhalation-based Anesthesia [87]	8
76	Hypertriglyceridemia, lipemia, and elevated liver enzymes associated with prolonged propofol anesthesia for craniotomy [88]	8
77	Late-onset rhabdomyolysis in burn patients in the intensive care unit [89]	8
78	Propofol Infusion Syndrome in Adult Cardiac Surgery [90]	8
79	Effects of Propofol on Cellular Bioenergetics in Human Skeletal Muscle Cells [91]	7
80	Propofol: Review of potential risks during administration [92]	7
81	Propofol infusion associated metabolic acidosis in patients undergoing neurosurgical anesthesia: A retrospective study [93]	7
82	Propofol-induced coved-type electrocardiogram during catheter ablation of paroxysmal atrial fibrillation: A case of Brugada syndrome? [94]	7
83	Attenuation of propofol tolerance conferred by remifentanil co-administration does not reduce propofol toxicity in rabbits under prolonged mechanical ventilation [95]	7
84	Propofol Use in Israeli PICUs [96]	6
85	Propofol-Related Infusion Syndrome: Rare and Fatal [97]	6
86	Drug-induced cardiovascular adverse events in the intensive care unit [98]	6
87	Intensive care unit delirium [99]	6
88	Lipid metabolism disturbances and AMPK activation in prolonged propofol-sedated rabbits under mechanical ventilation [100]	6
89	Case scenario: Tailored sedation to the individual needs of the intensive care unit patient [101]	6
90	Consider Heightened Awareness of Propofol Infusion Syndrome after Extracorporeal Membrane Oxygenation (ECMO) Decannulation [102]	5
91	Severe heart failure and rhabdomyolysis associated with propofol infusion in a burn patient [103]	5
92	Biochemical markers in total intravenous anesthesia and propofol infusion syndrome: A preliminary study [104]	5
93	Starvation-induced ketoacidosis in bariatric surgery: A case report [105]	5
94	Sedation and paralysis [106]	5
95	Recurrent Hyperkalemia During General Anesthesia in a Dog [107]	4
96	The Use of Propofol for Continuous Deep Sedation at the End of Life: A Definitive Guide [108]	4
97	A death associated with possible propofol infusion syndrome [109]	4
98	Clinical characteristics, treatments, outcome, and prognostic factors of severe autoimmune encephalitis in the intensive care unit: Standard treatment and the value of additional plasma cell–depleting escalation therapies for treatment-refractory patients [110]	3
99	Propofol toxicity in the developing mouse heart mitochondria [111]	3
100	The incidence of propofol infusion syndrome in critically-ill patients [11]	3

**Figure 1:**
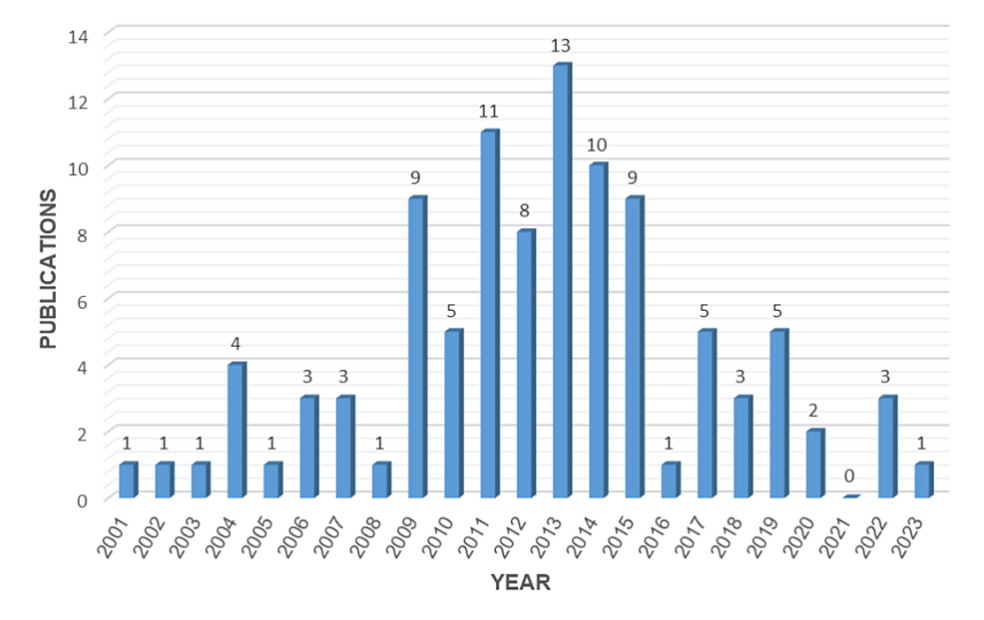
Number of publications on PRIS versus year PRIS: propofol-related infusion syndrome

In this analysis, the 100 most-cited articles on PRIS were published between the year 2001 and 2023. As shown in Figure 1, the majority of articles were published around the midway point of the 23 years.

Three journals, *Critical Care Medicine *(n=7), *Neurocritical Care* (n=6), and *Anesthesiology *(n=4) have the most publications. At least one paper, each from 72 different journals made it to the list of the top 100 most-cited articles. Further details are presented in Table 3.

**Table 3:** List of source journals

Rank	Journals	Number of publications
1	Critical Care Medicine	7
2	Neurocritical Care	6
3	Anesthesiology	4
4	Annals of Pharmacotherapy	3
5	Pediatric Critical Care Medicine	3
6	Respiratory Care	3
7	Journal of Anesthesia	2
8	Critical Care Nursing Quarterly	2
9	Acta Anaesthesiologica Scandinavica	2
10	Epilepsy and Behavior	2
11	Neurological Research	2
12	Epilepsy Research	2
13	Critical Care	2
14	Best Practice and Research: Clinical Anaesthesiology	2
15	Pediatric Nephrology	1
16	PLoS ONE	1
17	Current Treatment Options in Neurology	1
18	Current Neurology and Neuroscience Reports	1
19	Journal of Critical Care	1
20	BMJ Open	1
21	BMJ Case Reports	1
22	Therapeutic Drug Monitoring	1
23	Behavioural Pharmacology	1
24	Journal of Clinical Anesthesia	1
25	American Journal of Cardiology	1
26	Pediatric Radiology	1
27	Pediatrics	1
28	Anaesthesia and Intensive Care	1
29	Journal of Pharmacology and Pharmacotherapeutics	1
30	Muscle and Nerve	1
31	Journal of Clinical Medicine	1
32	Journal of Clinical Apheresis	1
33	Evidence-Based Child Health	1
34	Journal of Child Neurology	1
35	Injury	1
36	American Journal of Health-System Pharmacy	1
37	Journal of Cerebral Blood Flow and Metabolism	1
38	Pediatric Anaesthesia	1
39	American Journal of Kidney Diseases	1
40	Annals of Thoracic Surgery	1
41	Annals of Emergency Medicine	1
42	European Journal of Neurology	1
43	European Review for Medical and Pharmacological Sciences	1
44	Seminars in Respiratory and Critical Care Medicine	1
45	Danish Medical Journal	1
46	Journal of Neurosurgery: Pediatrics	1
47	Current Drug Safety	1
48	Frontiers in Veterinary Science	1
49	Indian Journal of Surgery	1
50	Journal of Pain and Palliative Care Pharmacotherapy	1
51	Acta Anaesthesiologica Belgica	1
52	Epilepsia	1
53	Clinical Toxicology	1
54	Electrolyte and Blood Pressure	1
55	Acta Pharmacologica Sinica	1
56	Journal of Cardiothoracic and Vascular Anesthesia	1
57	AANA Journal	1
58	Obesity Surgery	1
59	Journal of Korean Neurosurgical Society	1
60	Herzschrittmachertherapie und Elektrophysiologie	1
61	Journal of Surgical Research	1
62	American Journal of Therapeutics	1
63	New England Journal of Medicine	1
64	Neuropediatrics	1
65	Anesthesia and Analgesia	1
66	Association of Anesthetists	1
67	Lancet	1
68	Brain	1
69	Burns	1
70	Journal of Burn Care and Research	1
71	Journal of Allergy and Clinical Immunology	1
72	Pediatric Research	1

**Figure 2:**
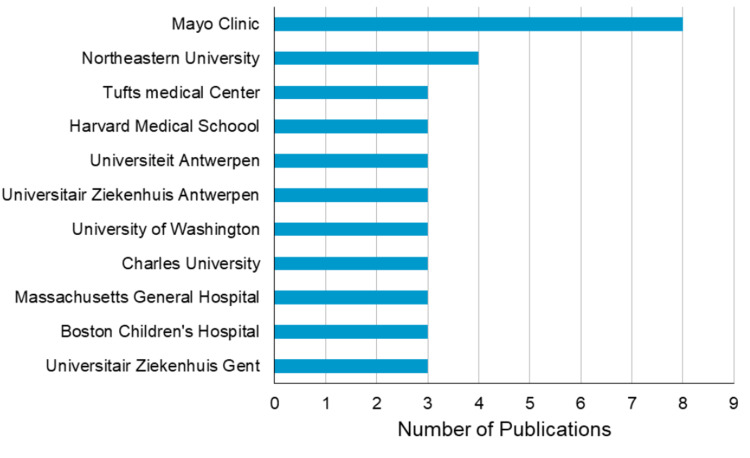
Top affiliated institutions contributing to PRIS research PRIS: propofol-related infusion syndrome

The Mayo Clinic was the top affiliated institution with the highest number of publications (n = 8). We limited our list to institutions that provided three or more publications. Further details are presented in Figure 2.

**Figure 3:**
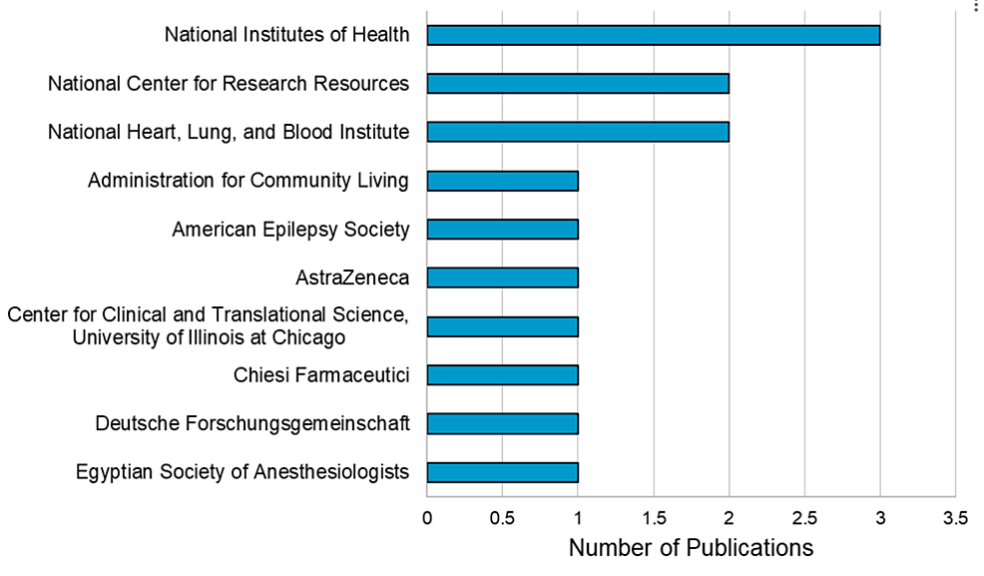
Top 10 funding sponsors for PRIS research PRIS: propofol-related infusion syndrome

More than 110 organizations contributed to research on PRIS. The top 10 funding sponsors of PRIS research are shown in Figure 3.

Many authors collaborated in the 100 most cited articles on PRIS research. We limited our list (Table 4) to those authors who contributed to two or more publications. Hocker, S. leads this list with four publications about PRIS.

**Table 4:** Top authors and their number of publications on PRIS PRIS: propofol-related infusion syndrome

Authors	Number of publications
Hocker, S.	4
Devlin, J.W.	3
Jorens, P.G.	3
Rabinstein, A.A.	3
Anděl, M.	2
Brophy, G.M.	2
De Paepe, B.	2
Duška, F.	2
Fong, J.J.	2
Hantson, P.	2
Krajčová, A.	2
Kälviäinen, R.	2
Okun, J.G.	2
Parviainen, I.	2
Ruthazer, R.	2
Schumaker, G.	2
Smet, J.	2
Sutter, R.	2
Van Coster, R.	2
Vanlander, A.V.	2
Waldauf, P.	2
Wijdicks, E.F.M.	2

With 47 publications, the United States has produced the greatest amount of literature on PRIS. Other contributing countries are listed in Table 5.

**Table 5:** Contributing countries and number of publications

Country	Number of publications
USA	47
Belgium	9
Germany	9
United Kingdom	5
Australia	4
Canada	4
Finland	4
Czech Republic	3
India	3
Israel	3
Netherlands	3
Switzerland	3
China	2
Denmark	2
France	2
Greece	2
Italy	2
Norway	2
South Korea	2
Bahrain	1
Brazil	1
Bulgaria	1
Croatia	1
Egypt	1
Japan	1
Lebanon	1
Malaysia	1
New Zealand	1
Portugal	1
Russian Federation	1
Saudi Arabia	1
Serbia	1
Sweden	1
Turkey	1

**Figure 4:**
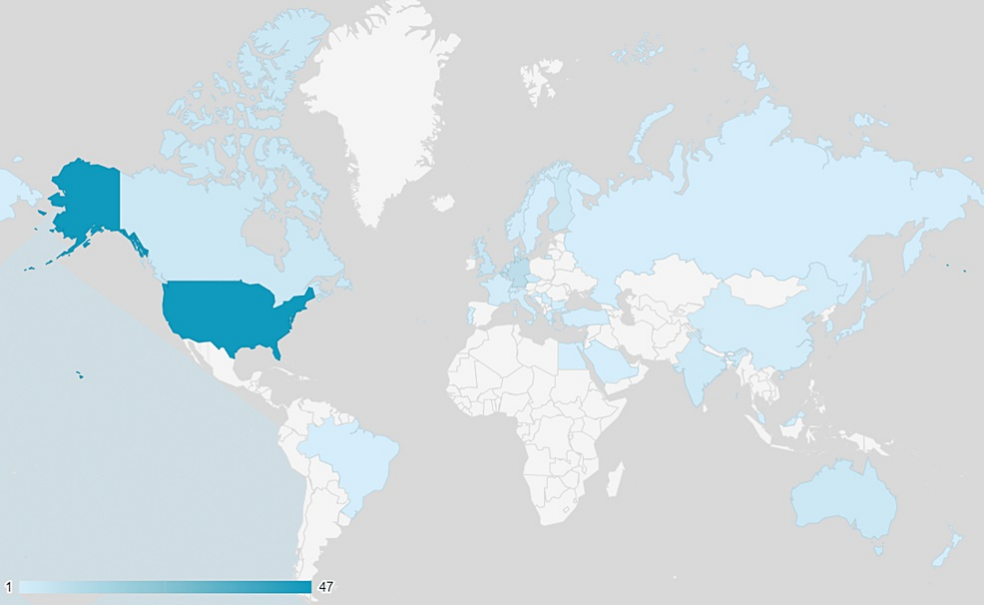
Geographic distribution of publications on PRIS PRIS: propofol-related infusion syndrome

Figure 4 presents the geographic dispersal of publications on propofol infusion syndrome on a global scale.

A review of the 100 most cited articles on PRIS published between 2001 and 2023 yielded common topics and themes, including toxic propofol infusion rates, pathophysiology, clinical manifestations, management, and mortality, as well as PRIS relation to refractory status epilepticus.

Discussions

Most Cited Article

We studied the top 100 publications on PRIS based on citation count for our analysis. “Propofol infusion syndrome” by Kam and Cardone (published in 2007) was cited in 364 instances, making it the most cited article. The authors wrote a thorough description of the clinical features, pathophysiology, and management of PRIS. Diagnosis criteria for PRIS include asystole secondary to acute refractory bradycardia with the addition of metabolic acidosis, rhabdomyolysis, hyperlipidemia, or enlarged fatty liver [13]. Based on the study conducted in this paper, PRIS is associated with infusion at doses higher than 4mg/kg/hr for a duration exceeding 48 hours. Young age, severe critical illness of central nervous system or respiratory origin, exogenous catecholamine or glucocorticoid use, depleted carbohydrate, and subclinical mitochondrial disease are potential predisposing factors to PRIS development [13]. With evidence from recent literature, Kam and Cardone proposed that a direct mitochondrial respiratory chain inhibition or impaired mitochondrial fatty acid metabolism mediated by propofol could be the cause of PRIS. Haemodialysis or haemoperfusion with cardiorespiratory support is the limited recommendation for treatment [13]. Overall, the paper aimed to educate and increase awareness of the rare but frequently fatal syndrome. The great citation value of this article may explain the growing debate on the appropriate use of propofol in anesthesia and intensive care units, as well as continuing attempts to understand the cause and identify treatment for the syndrome.

Publication Timeline

The top 100 most cited articles were published between 2001 and 2023. With the exception of the year 2021, at least one paper that was published each year throughout this time period made it to the list. This is most likely due to the lower accumulation of citation count in articles published in 2021 or the result of turmoil caused by COVID-19, as there was an elevated effort to put research emphasis on the global pandemic. The years 2013, 2011, and 2014 can be considered the most productive years, as they had the highest number of publications, 13, 11, and 10 respectively. The earliest article (published in 2001) reported a case of propofol infusion syndrome in a child who was successfully treated with haemofiltration. A significantly elevated plasma concentration of malonylcarnitine and C5-acylcarnitine prior to haemofiltration was normalized after recovery. The paper concluded that “abnormalities are consistent with specific disruption of fatty-acid oxidation caused by impaired entry of long-chain acylcarnitine esters into the mitochondria and failure of the mitochondrial respiratory chain at complex 11” [14]. This marks the beginning of our increased awareness of PRIS and the subsequent effort to study the syndrome. The most recent paper (published in 2023) investigated the standard treatment and additional escalating therapies for treatment-refractory cases of autoimmune encephalitis in the intensive care unit (ICU). PRIS was listed as a serious complication after six days of treatment in the ICU [110]. This explores other disease manifestations that are concomitant with PRIS.

Sources and Authors

Our selected 100 most cited articles were published in 72 different journals. The top three journals that contributed the most articles are *Critical Care Medicine* (n=7), *Neurocritical Care* (n=6), and *Anesthesiology *(n=4). Critical Care Medicine is ranked #3/100 (97th percentile) in the Critical Care and Intensive Care Medicine subcategory. *Neurocritical Care* is ranked #20/100 (80th percentile) in the Critical Care and Intensive Care Medicine subcategory, as well as #101/373 (73rd percentile) in the Neurology (clinical) subcategory. *Anesthesiology *is ranked #1/124 (99th percentile) in the Anesthesiology and Pain Medicine subcategory. The most common subject area or category covered by the top six journals is Medicine.

These 100 articles were published by 200+ different institutes and funded by 110+ different sponsors. Most notably, the Mayo Clinic was most involved and collaborated with eight publications on our list of most cited articles about PRIS. According to U.S. News & World Report’s 2023-2024 “Best Hospitals” rankings, the Mayo Clinic is top-ranked in more specialties than any other hospital and has been recognized as an Honor Roll member. They are working on more than 12,000 clinical studies, researching new treatments and therapies for the world’s most challenging and complex medical issues [112]. The National Institutes of Health (NIH) was the most generous sponsor and contributed to three publications. Overall, the NIH “invests approximately $45 billion annually in medical research for the American people” [113].

More than 250 different authors contributed to the 100 most cited articles on propofol infusion syndrome. The top authors are Sara E. Hocker (n=4), John W. Devlin, (n=3), Philippe G. Jorens (n=3), and Alejandro A. Rabinstein (n=3). According to the Elsevier Health website, “Dr. Sara E. Hocker, MD is Associate Professor of Neurology, Mayo Clinic College of Medicine; Director, Neurocritical Care Fellowship; Consultant, Neurosciences Intensive Care Unit, Mayo Clinic Hospital, Saint Marys Campus, Rochester, Minnesota. She is the author of more than 60 publications in her field” [114].

Overall, the highly ranked journals, the affluent institutions, the generous donors, and the prominent authors that collaborated in the study of PRIS suggest the potential rise in frequency and popularity of the syndrome.

Collaborating Countries

Among the 34 participating countries, the United States has the highest number of publications (n=47). As of 2015, “the US is still the source of 44% of the world’s medical research funds, with Europe at another 33%” [115]. In 2020, “Americans are usually the first to gain access to major new medical advances, advances often discovered at American universities and developed by American companies. As a result, the U.S. ranked first for both Choice and Science & Technology” [116]. In addition, the medical and health research and development investment in the U.S. also reached $245.1 billion in 2020, which is an 11.1% increase from 2019 [117]. This explains why the USA leads our list with approximately five times more publications than the countries with the second most publications, Belgium (n=9) and Germany (n=9).

Toxic Propofol Infusion Rates

Several studies sought to elucidate the rates at which dose-related propofol infusion manifests with symptoms of toxicity. A literature review of case reports and small case series by Kang et al. concluded that caution should be exercised when infusing propofol in critically ill patients at rates of >5 mg/kg/hour and over a time period of >48 hours [21]. An article by Short et al. yielded similar recommendations, with evidence supporting the presence of acute cardiomyopathy and skeletal myopathy when propofol is infused at rates of 5 mg/kg/hour and greater for more than 48 hours [29]. An article by Koriyama et al. analyzing propofol infusion practices at a single-center referral pediatric intensive care unit also concluded that safe infusion can be ascertained when limiting doses to 4 mg/kg/hr and for less than 24 hours, in agreement with previous recommendations [58].

However, other articles have suggested infusion rates at which PRIS begins to manifest are actually lower than previously presumed. A highly cited study by Krajčová et al. reviewing 153 case reports and experimental studies on PRIS pathophysiology revealed that propofol infusion syndrome can develop at infusion rates as low as <4 mg/kg/h, albeit different types of PRIS-associated symptoms emerge at varying infusion rates [19]. For example, the same study by Krajčová et al. found that metabolic acidosis occurs sooner after infusion begins, whereas more serious symptoms of arrhythmias develop when infusion duration surpasses 48 hours and rhabdomyolysis and hyperlipidemia manifest after 96 hours. Additionally, the rare incidence of PRIS at rates traditionally considered safe for ICU patients is highlighted in a case report by Annecke et al. studying a 36-year-old patient with a severe head injury who suffered mortality from PRIS even at a moderate rate of 2.8 mg/kg/hr, well within the recommended limits [80]. Such cases emphasize the importance of exercising caution when resorting to continuous propofol infusion in critically ill patients, given their already complicated clinical background such as high endogenous myocardial catecholamine levels and concurrent vasopressor use in patients presenting with severe head trauma.

Pathophysiology of PRIS

Our bibliometric analysis found various articles that studied the pathophysiologic mechanisms behind PRIS, a diagnosis largely based on clinical signs and lab findings. The most commonly cited article in our analysis, by Kam et al., highlighted the mechanisms and pathophysiological features of this condition [13]. PRIS has been found to be associated with impaired mitochondrial respiratory chain function, ultimately leading to failure of adenosine triphosphate (ATP) production and metabolic acidosis. Furthermore, disruption of mitochondrial fatty acid oxidation and lipid metabolism leads to a toxic build-up of fatty acid intermediates in serum, cardiac, and skeletal muscle, contributing to manifestations of ventricular arrhythmias and worsening metabolic acidosis, especially during states of increased stress such as starvation and infection [13]. Another article by Vanlander et al. investigated the molecular mechanisms behind PRIS by experimental sedation of rat models which yielded conclusions further emphasizing the dysfunction of mitochondria at the main site of interaction with propofol, particularly from the impediment of electron flow through the respiratory chain and coenzyme Q [26]. Other studies further support this theory that PRIS results from inhibition of mitochondrial oxidative phosphorylation and lipid metabolism, particularly during states of increased metabolic demand with reduced glycogen reserves [14,35-38,48,100,111]. Of note, various studies of histological and electron microscopal analysis of liver, skeletal, and heart muscle in autopsies from PRIS patients demonstrated findings of electron-dense bodies in liver and muscle cells associated with free fatty acid accumulation [66,91].

Commonly Reported Clinical Manifestations of PRIS

Several articles included in our bibliometric study called attention to various clinical manifestations that have been commonly reported for PRIS. A widely cited prospective study by Roberts et al. analyzed data from critically ill adults from 11 academic medical centers who were prescribed propofol for more than 24 hours to identify the incidence of PRIS [17]. The data from this investigation demonstrated that the earliest signs of PRIS are new-onset metabolic acidosis and cardiac dysfunction, followed by new-onset rhabdomyolysis, renal failure, and hypertriglyceridemia [17]. Indeed, several case studies accentuated the early clinical manifestations of lactic acidosis following propofol infusion, supporting the previously mentioned theories of PRIS-related mitochondrial dysfunction [28,32,34,53,61,68]. Thereafter, symptoms of cardiac dysfunction start to materialize, often evidenced by ECG abnormalities such as tachycardia and T-wave inversions [62,70,83]. Several studies have also found ECG changes of Brugada-type ST elevations to be associated with imminent fatality [94,103]. These articles have accentuated the clinical usefulness of ECG monitoring for potential cardiac toxicity when infusing large doses of propofol for extended periods of time.

Other clinical manifestations of PRIS include rhabdomyolysis, renal failure, and hypertriglyceridemia. Numerous corroborative studies reinforced the notion that rhabdomyolysis manifests later in the course of propofol infusion, especially in critical care settings [24,89,103]. The observations from this research delineated the need for special attention when administering propofol for burn patients as they are more prone to rhabdomyolysis due to the nature of their injury. Interestingly, a small study by Lehavi et al. which examined the effects of propofol anesthesia on rhabdomyolysis markers in obese patients undergoing gastric bypass surgery found no significant difference in rhabdomyolysis incidence when comparing propofol-based anesthesia to inhalational-based anesthesia [87]. Thus, further research is required to elucidate how various patient factors may affect this relationship. Related to rhabdomyolysis is acute renal failure. A case report by Casserly et al. features two patients who sustained renal failure following propofol infusion manifesting as elevated creatinine [33]. Another article by Linko et al. describes the case of an adult burn patient who developed renal failure following low-dose propofol infusion but was successfully stabilized following continuous renal replacement therapy and cessation of infusion [103]. Hypertriglyceridemia is also widely acknowledged to be associated with propofol infusion syndrome [59,63,88]. Notably, one study performed at an academic medical center in New York showed that elevated triglyceride concentrations occurred more often and at lower doses in mechanically ventilated patients with concurrent COVID than those without COVID, in the setting of continuous propofol infusion [84].

These articles highlight the importance of close clinical monitoring for signs of PRIS such as from the time that propofol infusion is initiated, regardless of dose for the entire duration of therapy.

Management of PRIS

Various articles included in our bibliometric study provided important insight into the management of PRIS. As PRIS is largely a clinical diagnosis, critical lab markers assist in monitoring both the risk and progression of PRIS. A study by Schroeppel et al. highlights the importance of screening for PRIS by obtaining serum creatine phosphokinase levels [60]. Previous literature and articles from this review share a consensus that the most supported intervention for PRIS is the cessation of propofol infusion. For example, a case report by Liolios et al. featured PRIS during short-term large-dose propofol infusion in a neurosurgical procedure followed by small-dose infusion postoperatively [24]. In this case, new-onset lactic acidosis, renal failure, and abnormal rhabdomyolysis markers were reversed shortly after propofol was withdrawn. Several articles similarly emphasize successful stabilization of patient symptoms and abnormal lab values in patients with PRIS following discontinuation of infusion [25,34,41,42,43,51,90,92]. Additional management includes consideration of substituting propofol with alternative sedating agents such as alfentanil and midazolam [35,99,106]. Other novel therapies have been proposed to significantly improve PRIS symptoms, such as therapeutic plasma exchange proposed by case reports from Levin et al. and Da-Silva et al. [74,81]. The utility of future investigations into alternate treatment for PRIS is paramount to tailor patient-specific remedies and maximize patient welfare.

PRIS and Status Epilepticus

Propofol infusion is a mainstay treatment option for refractory status epilepticus (RSE), and thus PRIS should be considered as a potential complication associated with significant morbidity and mortality when managing RSE patients [20,64,72,75]. Its effectiveness at controlling seizures in the critical care setting is well studied, particularly in comparison with other sedatives [15,16,31]. However, caution should be exercised when perfusing at high doses for longer than 48 hours, as suggested by Parviainen et al. and Power et al. [27,35]. Of note, several studies alluded to the increased propensity for PRIS in RSE patients who are on long-term steroids and/or in the setting of catecholamine release [30,39]. In the critical care setting, a retrospective cohort study by Hawkes et al. determined that the use of anesthetic drugs for seizure control in status epilepticus patients was not associated with in-hospital or 90-day mortality, however, did prolong hospital stay [56]. Beyond RSE, PRIS can also present in acutely ill neurocritical care patients as propofol can be used for emergency neurological life support [79]. Therefore, consideration of the additional risks against targeted benefits should direct clinical decision-making when administering propofol to RSE patients, as this anesthetic agent can unintentionally worsen outcomes [45].

Mortality from PRIS

In our analysis, there were many reported cases of mortality associated with PRIS that draw attention to the complexity and gravity of this condition. Fong et al. performed a retrospective analysis and confirmed the increased risk of mortality associated with various previously mentioned predictive factors of suspected PRIS [17]. A study by Veldhoen et al. highlights the fatal case of PRIS in a 17-year-old patient who was frequently monitored with blood gases, serum lactate, and creatine kinase, suggesting that clinical monitoring with laboratory studies may create a false sense of security and should not be considered sufficient in preventing PRIS [65]. This indicates the need for future research into adjunct methods of monitoring symptom severity of PRIS. Another article by Baumeister et al. reports the case of PRIS-associated mortality in a pediatric patient who developed the syndrome after a ketogenic diet was initiated, thus pointing to the complexity of pharmacodynamic interactions that may arise [23]. Another case of a previously healthy young trauma patient describes the progression of PRIS following infusion of 1.4-5.1mg/kg/h for 88h to multiorgan failure and rhabdomyolysis [44]. Cardiopulmonary arrest and hyperkalemia have also been reported as succeeding propofol infusion, resulting in eventual mortality [67,76,109]. This suggests important implications for cardiac involvement. Given the multiplicity of complications associated with propofol infusion, case reports remain a pragmatic source of literature to highlight clinically challenging instances of PRIS.

## Conclusions

This bibliometric analysis serves to highlight trends in research on PRIS. Among the top 100 most cited articles, frequently discussed subtopics include toxic infusion rates and duration, clinical manifestations, management, and mortality due to PRIS. While the effect of high-dose propofol on the mitochondrial respiratory chain is generally agreed upon, the infusion rate and duration of the anesthetic remain central to the debate behind the onset of PRIS. As most literature to date is limited to case reports and retrospective studies, recommendations and evidence based on different patient contexts and ICU durations lead to discrepancies in the threshold at which researchers observe the symptoms of PRIS. This lack of uniformity may be attributed to the rarity of the condition and uniqueness of each case report. These reports highlight the importance of close clinical monitoring beyond just lab values for signs of PRIS for the entire duration of infusion, regardless of dosage. The most supported intervention is cessation of infusion, though other novel therapies such as plasma exchange have been noted to significantly improve symptoms. The utility of future investigations into alternative or adjuvant treatments for PRIS is critical to tailoring patient-specific remedies and maximizing welfare. Though rare in occurrence, the many reports of mortality associated with the condition draw attention to its gravity and complexity. This bibliometric analysis ultimately illustrates the global trends in publications on PRIS, providing an overview of the evolving practices, management, and understanding of the phenomenon in the fields of critical care, neurology, and anesthesiology.
